# Understanding cystic lung lesions in smokers with interstitial lung disease: radiologic–pathological correlation

**DOI:** 10.1186/s13244-025-02074-7

**Published:** 2025-09-17

**Authors:** Juan José Arenas-Jiménez, Ignacio Aranda, Svetlana Shalygina, Cristina Alenda, David Ferrandez-Ferrandez, Elena García-Garrigós

**Affiliations:** 1 Department of Radiology, Hospital General Universitario Dr. Balmis de Alicante, Alicante, Spain; 2 Alicante Institute for Health and Biomedical Research (ISABIAL), Alicante, Spain; 3 Department of Pathology and Surgery, Miguel Hernández University, Alicante, Spain; 4 Department of Pathology, Hospital General Universitario Dr. Balmis de Alicante, Alicante, Spain

**Keywords:** Smoking, Emphysema, Interstitial lung disease, Pulmonary fibrosis

## Abstract

**Abstract:**

Due to destructive, fibrotic, and remodeling mechanisms, we can find a varied constellation of aerated and cystic lung lesions in smoker patients with interstitial lung disease that pose a diagnostic challenge for both radiologists and pathologists. Radiologic terminology used for cystic lung lesions in smokers is varied and sometimes confusing, and the same applies to their pathologic correlation, with different names for similar findings. Moreover, there is substantial overlap among different cystic lesions in both radiology and pathology. Ultimately, the diagnosis of a given type of cyst may lead to a wrong diagnosis with important clinical implications. In this setting, the goals of this article are to present a diagnostic approach to these lesions by correlating radiologic findings with pathology and describing a series of radiologic characteristics of these lesions, which we have called “the four S of cystic lung lesions in smokers” for size, site, shape, and surrounding of the lesions. We will define the clue radiological findings of centrilobular emphysema, paraseptal emphysema, thin-walled cysts, traction emphysema, honeycombing, smoking-related diffuse cystic lung disease, cysts in Langerhans cell histiocytosis, and cystic lesions appearing in desquamative interstitial pneumonia and we will try to show a correlation of each of these lesions with pathology for a better understanding of radiological findings. Finally, we will deal with fibrosing lung diseases and cystic lung lesions in smokers, specifically with smoking-related interstitial fibrosis and its pathological variants, and with usual interstitial pneumonia, whose prognosis is strikingly different.

**Critical relevance statement:**

Knowledge of the pathological correlation of the different cystic lesions that appear in smokers with interstitial lung disease permits a better understanding of their radiological manifestations.

**Key Points:**

- Interstitial lung disease in smokers is characterized by varied cystic lung lesions.
- Cystic lesions are characterized by their size, site, shape, and surroundings.
- Cystic lesions in smokers may help to characterize the underlying fibrosing disease.

**Graphical Abstract:**

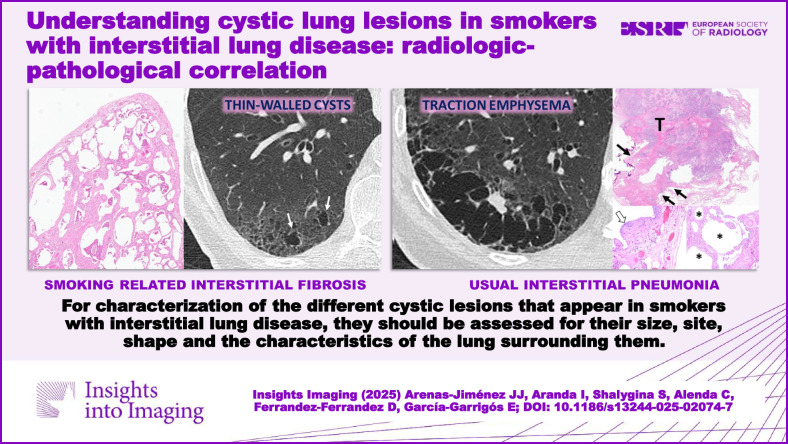

## Introduction

Interstitial lung disease (ILD) in smokers is the result of a combination of inflammatory, destructive, organizing, and fibrotic phenomena that manifest radiologically as a spectrum of increased attenuation of the lung combined with destructive aerated lung lesions, whose appearances are sometimes difficult to differentiate from each other [1].

Pathologically, there is a frequent overlap of findings and conditions in the lungs of smokers. This makes both radiological and pathological literature on ILD in smokers sometimes confusing and even misleading, with several names for similar radiological findings or pathological diagnoses [1–6].

This article aims to present the radiological spectrum of aerated lesions that can be found in smokers, paying attention to their distinctive radiological characteristics and showing a correlation of these lesions with pathology for a better understanding of what radiological findings represent. A deep discussion of what radiologists see, and how it correlates with pathology, should be presented based on the authors‘ experience with radiologic–pathological correlation of ILD in smokers and through a review of the literature. This could aid the understanding of the complex spectrum of ILD in smokers and specifically of aerated lesions that range from merely destructive lesions lacking perceptible walls, such as emphysema, to truly cystic lung lesions whose interpretation is sometimes challenging. So, we will apply the term “cystic lung lesion” in a broad sense to make reference to a constellation of aerated lesions occurring in the lungs of smokers.

The description of the radiologic characteristics of the different types of cystic lesions, followed by their pathological counterpart, are shown in Tables 1 and 2.

**Table 1:** Review of CT characteristics and pathological findings of cystic lung lesions associated with ILD in smokers

Cystic lesion	Size of the lesion	Size of the walls*	Site	Shape	Surroundings	Main pathological findings of the cysts
Centrilobular emphysema	Variable, from a few mm up to 3 cm May be confluent	Lacks visible walls	Anywhere throughout the lung, but usually do not abut the pleura Upper lobe predominance	Variable, more or less rounded, although ill-defined Larger lesions may be polygonal Central dot due to the centrilobular artery	Normal lung or more emphysema Frequent centrilobular GGO due to respiratory bronchiolitis May accompany any other ILD	Destruction of alveoli without obvious fibrosis
Paraseptal emphysema	Variable, from a few mm to more than 1 cm	Thickened septa are visible as a thin, regular line	Subpleural, single-layer, always limited by some pleural surface	Roughly polygonal, limited by thickened interlobular septa perpendicular to the pleural surface and frequently separating emphysematous from normal lung centrally	Normal lung or more emphysema May accompany any other ILD	Peripheral airspaces are separated by thickened fibrous septa
Thin-walled cysts	Around 1 cm and irregular in size	Thicker than in emphysema but thinner than in other cysts, such as those from honeycombing or traction emphysema “Less than 1 mm”	Next to pleural surfaces, but usually not abutting the pleura Middle/upper zones of the lungs	Irregularly shaped	Can be a normal lung, but frequently a faint ground-glass and reticulation as a manifestation of accompanying SRIF and DIP	Emphysematous areas surrounded by collagen fibrosis
Traction emphysema	Initially can be small but usually coalesce, forming big cystic lesions	Interrupted thick septa	Extends along the pleural surface as paraseptal emphysema Frequently, in the posterior aspect of the lower lobes	Lobulated appearance Interrupted septa showing the “stalactite and stalagmite sign” appearance	Traction bronchiectasis may appear in the periphery of the lesions	Dense fibrosis with occasional fibroblastic foci
Honeycombing	Quite variable, from “microcystic” honeycombing below the resolution of CT scanners to more than 2 cm Usually uniform size of cysts	Relatively thick	Usually several rows, pleural-based Frequently basal posterior	Clustered, rounded cysts sharing walls	Lung architectural distortion Associated with traction bronchiectasis	Enlarged airspaces surrounded by fibrosis and lined by bronchiolar or hyperplastic alveolar epithelium
Smoking-related cystic lung disease	Variable size from 2 mm to 2 cm	Relatively thin	Diffuse, away from the pleura Frequently perivascular	Rounded to oval	Normal lung or other findings related to smoking, such as emphysema	Airspaces surrounded by alveolar walls of normal thickness
Cysts in Langerhans cell histiocytosis	Variable, from a few mm to 2–3 cm	Relatively thick	Diffuse, frequent peribronchial Spares costophrenic angles and medial tips of the lingula and the middle lobe	Irregular Bizarre appearance Cavitated nodules	Stellated nodules Cavitated nodules	Fibrotic walls. Diagnosis is made by detecting aggregates of Langerhans cells
Cysts in DIP	Tiny 2–4 mm	Thin	Diffuse Basal predominance	Round	Diffuse basal ground-glass and reticulation	Fibrotic cysts Dilatation of alveolar ducts and bronchiectasis

*mm* millimeters, *GGO* ground-glass opacities, *ILD* interstitial lung disease, *SRIF* smoking-related interstitial fibrosis, *DIP* desquamative interstitial pneumonia

* Measurements of walls’ thickness are relatively inaccurate at this size level, and they are variable even in the same category of cysts, for that reason, categorization of the size of the walls is made in a comparative way among the different types of cysts

**Table 2:** Distribution of some characteristics of the cystic lesions among different types of cysts

Characteristics of the lesion	Type of cysts associated with this finding
Site	
Subpleural	Paraseptal emphysema Honeycombing Traction emphysema
Peripheral not abutting the pleura	Thin-walled cysts
No specific relation to the pleura	Centrilobular emphysema Cyst associated with Langerhans cell histiocytosis Smoking-related cystic lung disease
Shape	
Rounded	Honeycombing Cysts in desquamative interstitial pneumonitis Smoking-related cystic disease
Irregular	Traction emphysema Thin-walled cysts Cyst associated with Langerhans cell histiocytosis

## The four Ss of cystic lung lesions in smokers

A number of characteristics must be checked for a better radiologic categorization of each cystic lesion. We have dubbed these “the four S of cystic lung lesions in smokers”: size, site, shape, and surrounding.

Size refers to the size of the cysts themselves and the thickness of their walls, which is an essential feature. They can be uniformly or variably sized. The thickness of the walls is difficult to categorize and measure. As will be discussed moving forward, it can range from absent walls to less than 1 mm and thicker than 1 mm, but the most useful information is provided by the comparison of the wall thickness along the different types of cysts.

The site of the cystic lesion related to the pleural surface is a key finding. They can be touching the pleura, be peripheral, not abutting the pleura, or be away from the pleural surface.

The shape of the lesions is also an important distinguishing feature. Cysts can be round, polygonal, or irregular.

Finally, radiologists should be aware that a single finding, in this case a type of cyst, does not necessarily make a diagnosis. For this reason, the characteristics of the lung surrounding the cysts may be a cornerstone finding for diagnosis.

## Radiologic cystic lung lesions in smokers

### Centrilobular emphysema (CLE)

**Fig. 1:**
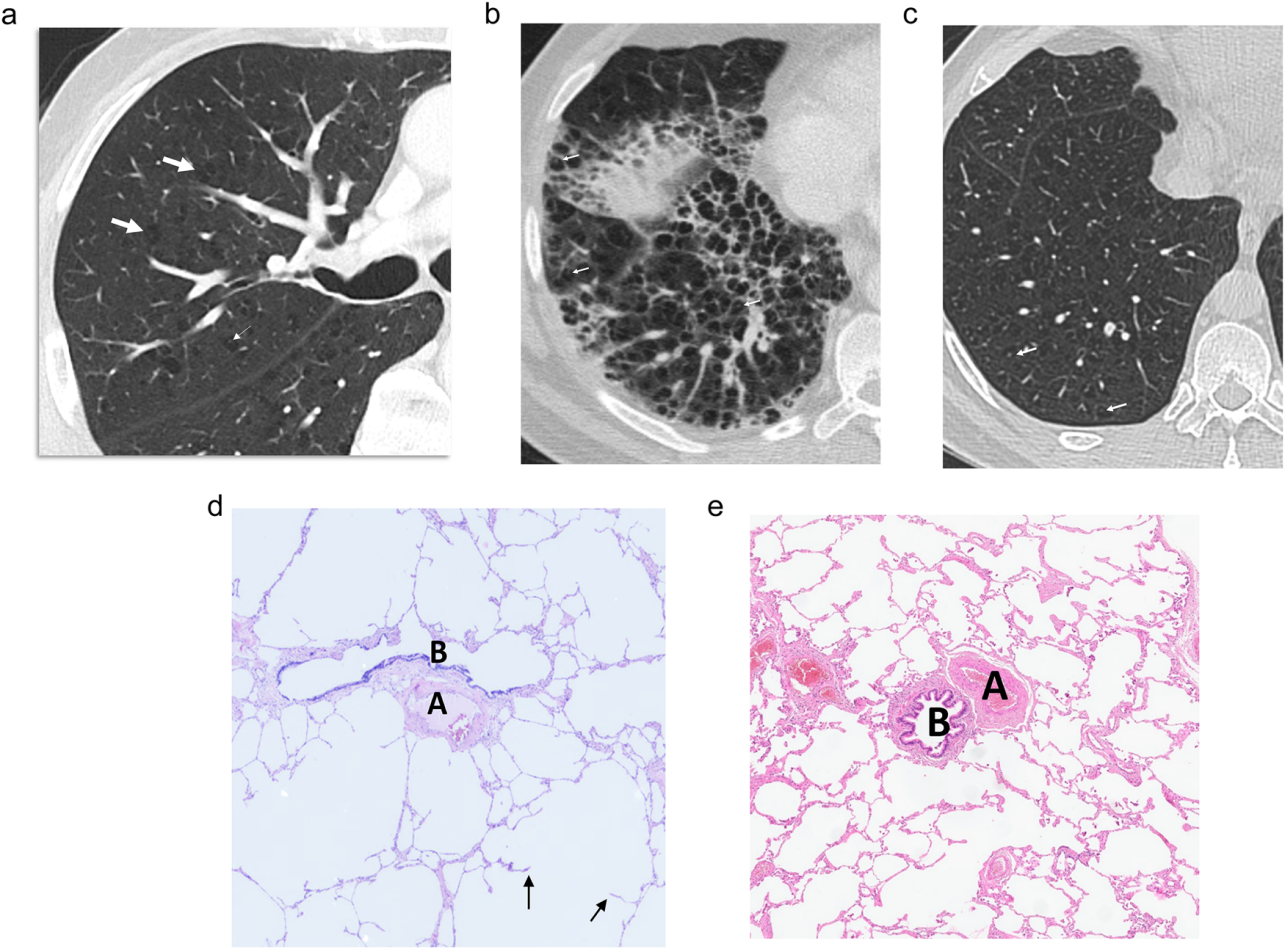
CLE. **a** CT shows CLE as small, rounded areas of low attenuation without defined walls surrounded by normal lung (arrows). Central dots are seen in some lesions (small arrows). **b** CT in a 56-year-old male smoker admitted for chronic obstructive pulmonary disease exacerbation with fever. Multiple rounded cysts are seen corresponding to emphysema with thickened inflammatory walls. Their appearance may mimic honeycombing, but central dots (arrows) allow differentiation. **c** CT scan of the same patient 1 month after b showed CLE without defined walls and the central dot in many of the lesions (arrows). **d**, **e** In the photomicrographs corresponding to other patients, compare the number and size of alveoli seen in the normal lung (**e**) with the emphysematous lung (**d**). In both cases, a bronchiolar structure (B) and the centrilobular artery (A) are seen, the latter corresponding to the central dot visible at CT

CLE at CT manifests as non-peripheral small rounded areas of low attenuation surrounded by normal lung without defined walls [7–9]. Therefore, strictly speaking, CLE does not appear as cysts. When CLE extends, it spans several secondary pulmonary lobules that form bigger confluent lesions, and sometimes exhibits a polygonal shape with no walls or is limited by interlobular septa [8]. The recognition of the centrilobular artery as a central dot in these areas is a helpful finding (Fig. 1a). “Simple emphysema” lacks definable walls. However, inflammation or edema can lead to the appearance of defined walls that may mimic other cystic lesions (Fig.1b, c). CLE predominates in the upper lobes and its size ranges from less than 1 mm to more than 3 cm [8].

Histologically, CLE is defined as permanent, abnormal enlargement of the respiratory airspaces, accompanied by destruction of their walls without obvious fibrosis [10]. It results from inflammation of the respiratory bronchioles [11, 12]. The so-called “simplification of the alveolar structure” due to destruction of alveoli is seen (Fig. 1d, e). The walls of areas of emphysema are lined by fragmented alveolar epithelium, but frequently, recognition of some grade of fibrosis is acceptable.

### Paraseptal emphysema (PSE)

**Fig. 2:**
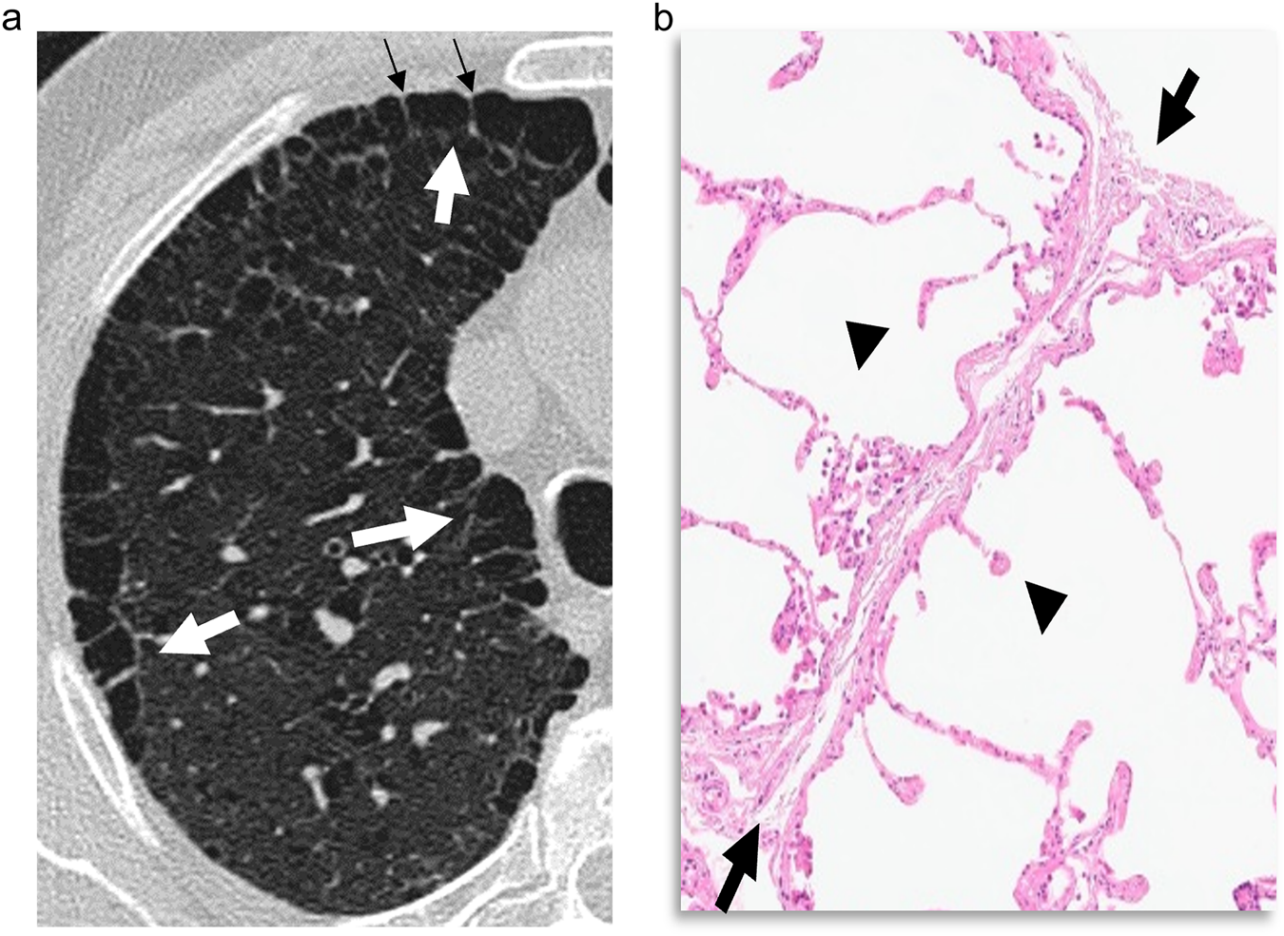
PSE. **a** CT shows foci of well-defined low attenuation areas (white arrows) separated by intact interlobular septa (small black arrows), which are thickened. They are subpleural in the costal and mediastinal pleural surfaces. **b** The photomicrograph in another patient shows dilated airspaces due to destruction of alveolar walls, depicted by some free-floating alveolar wall fragments (arrowheads), and affecting the subpleural area and next to an intact interlobular septa (arrows)

The term PSE was coined for emphysema involving the distal acinus [13]. Lesions tend to occur adjacent to interlobular septa and beneath the pleura, and most frequently in the upper lobes [13]. It exhibits a characteristic appearance at CT consisting of foci of low attenuation separated by intact interlobular septa that are thickened (Fig. 2a).

PSE is distributed along the pleural surfaces of the peripheral lung, the fissures, and the mediastinal pleura, mainly in the upper lung, but it also appears along the peribronchovascular spaces, usually as rows of emphysematous lesions sharing their walls [8, 13]. It is of note that PSE is the most frequent type of emphysema in the syndrome of combined pulmonary fibrosis and emphysema (CPFE) [14, 15].

Pathologically, PSE is seen as peripheral airspaces separated by thickened septa (Fig. 2b) and associated septal veins leaving thin filaments to bridge the gaps [13]. Occasionally, a row of small foci of emphysema are seen that may mimic honeycombing, however, the lack of architectural distortion and other signs of fibrosis distinguish both lesions.

### Thin-walled cysts

**Fig. 3:**
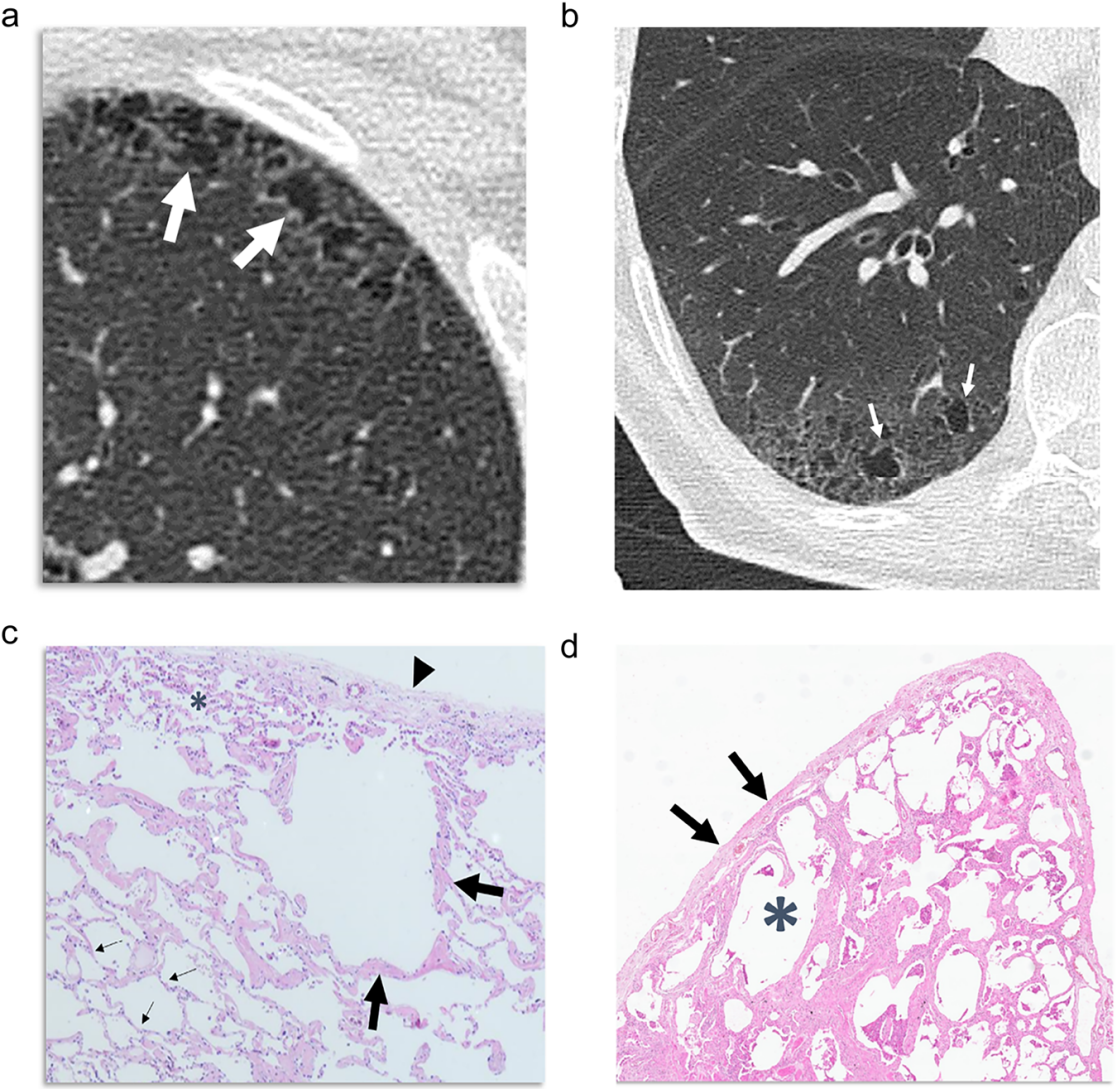
Thin-walled cysts are shown in CT of two different patients with SRIF, located in the periphery of the anterior upper lobe (**a**) and the dorsal aspect of the lower lobe (**b**), slightly apart from the pleural surface. They show irregular shapes and are associated with faint reticulation. **c** Pathologic specimen of the left upper lobe of the patient in **a**: these cysts represent emphysematous areas surrounded by collagen fibrosis (black arrows) compared with “simple emphysema,” whose walls are thinner non-fibrotic alveolar walls (thin arrows). Subpleural fibrosis (arrowhead) and pigmented macrophages extending into the airspaces, forming DIP-like areas (asterisk), are seen. **d** Lung biopsy of the right lower lobe of the patient in **b** showed dense eosinophilic ropey-appearing collagen thickening the alveolar septa and limiting the dilated airspaces. Subpleural fibrosis (arrows) separates dilated airspaces (asterisk) from the pleural surface

Thin-walled cysts were first defined by Watanabe et al [16] as a radiologic characteristic of airspace enlargement with fibrosis (AEF). AEF was described as a frequent finding in specimens of lobectomy for lung cancer [17, 18], and it has different pathologic findings compared with CLE and usual interstitial pneumonia (UIP) that will be discussed [19]. The multiple thin-walled cysts seen in AEF show a subpleural distribution, although they do not abut the pleura (Fig. 3a, b). They are variable sized (less than 15 mm in patients with AEF alone), with thin walls (regarded as less than 1 mm in thickness, although measurements at this range are limited), and they are not distributed along the lung base, but in the upper lobes and the upper and middle portion of the lower lobes [16]. Similarly, Otani et al [17] described that smoking-related interstitial fibrosis (SRIF) with pulmonary emphysema was associated with clustered cysts with visible walls that showed a markedly irregular size and shape, thin walls, and relatively less involvement of the subpleural parenchyma. Recently, in a series of 23 patients with pathologically confirmed SRIF, multiple thin-walled cysts were present in 73.9% of the patients with SRIF, compared with 2% of patients with emphysema and none with UIP [20].

Pathologically, these cysts represent enlarged airspaces surrounded by collagen fibrosis (Fig. 3c, d) compared with the “simple emphysema,” whose walls are thinner and non-fibrotic, fragmented alveolar walls [19]. Some authors argue that most cases of AEF are actually CLE with fibrosis [2], since smoking commonly produces a degree of fibrosis in the walls of the respiratory bronchioles, and this fibrous tissue may extend around the enlarged airspaces of CLE, which in turn is caused by damaged bronchioles. The term “emphysematous fibrosis” is used by other authors [21]. Subpleural fibrosis separating these cysts from the pleura accounts for the localization of these lesions slightly apart from the pleural surface (Fig. 3c, d), frequently with intervening ground-glass attenuation [20].

### Traction emphysema

**Fig. 4:**
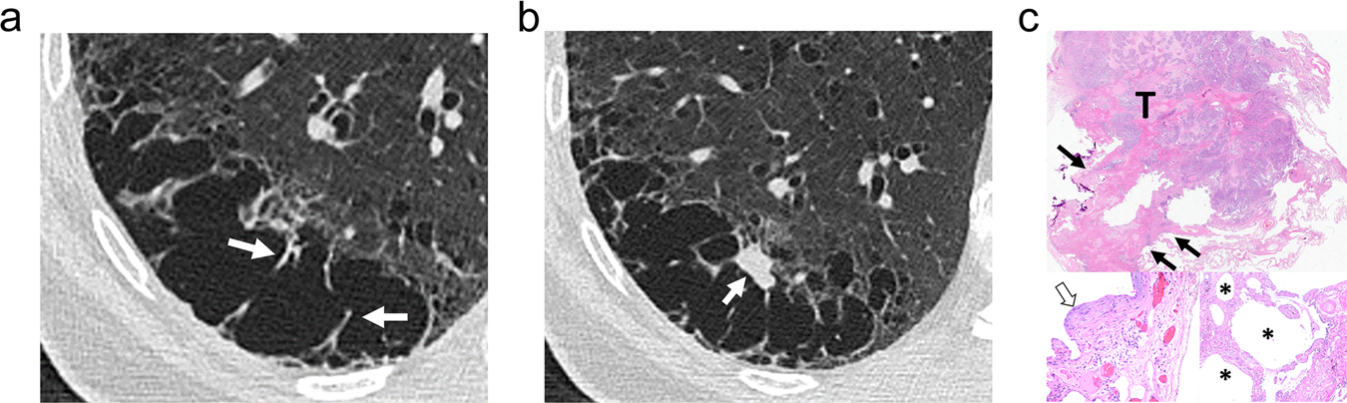
Traction emphysema lesions in a 62-year-old male smoker who underwent a right lower lobe lobectomy for lung cancer. **a** CT shows irregular cystic lesions extending along the pleural surface exhibiting interrupted septa in their walls, described as the “stalactite and stalagmite sign” (arrows). **b** A lung nodule (arrow) is seen in the wall of this traction emphysema cyst. At pathology (**c**), dense collagen fibrosis (black arrows) next to the tumoral lesion (T) is seen. Away from these lesions, there were findings of a UIP pattern with fibroblastic foci (open arrow) and honeycombing cysts (asterisks)

**Fig. 5:**
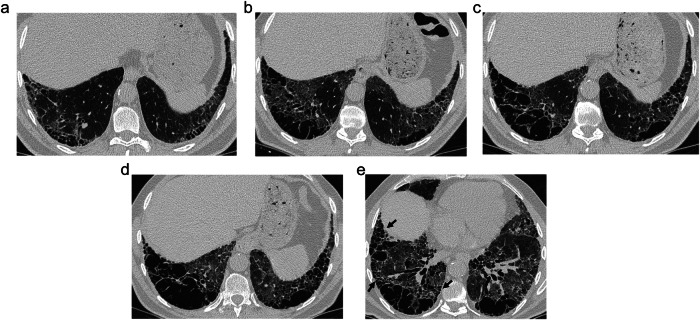
Radiological evolution of traction emphysema in a male smoker who was 59-years-old at the time of the first scan. **a**–**d** Yearly CT scan follow-up during a period of 4 years shows progression of bilateral basal subpleural cystic lesions with progressive enlargement and confluence. In **e**, honeycombing and traction bronchiectasis are seen in the vicinity of the traction emphysema lesion (arrows). The patient developed a severe progressive deterioration in exercise capacity

Traction emphysema lesions are named after the Official ATS/ERS/JRS/ALAT Research Statement published in 2022 on the Syndrome of CPFE [1]. Previously, they had been regarded as “thick-walled cysts” by Inomata et al [22], who described them as associated with CPFE syndrome in the form of combined emphysema and idiopathic pulmonary fibrosis. The name “traction emphysema” refers to a similar pathogenic mechanism to the traction bronchiectasis seen in fibrosis. These cysts have been deemed a characteristic phenotype of CPFE, appearing in patients with both “idiopathic” and connective tissue disease-related CPFE [5, 14, 15, 23], mostly associated with a UIP pattern. However, recent articles make reference to these cysts as a possible manifestation of SRIF [1, 4, 24, 25]. In the original article by Inomata et al [22], they appeared in the upper and lower lobes with a similar frequency, in a subpleural location. Their walls are thicker than those in thin-walled cysts (> 1 mm), and they typically exhibit interrupted thick septae (Fig. 4a, b), giving the characteristic appearance of the so-called “stalactite and stalagmite sign” [4]. Sometimes, bronchi that open to the cystic space can be seen on their walls. Although they can initially be small, they frequently grow and coalesce to form big cystic lesions extending along the pleural surface (Fig. 5). Other signs of a UIP pattern, such as traction bronchiectasis and honeycombing, are frequent, and in our experience, they may appear as a phenotypic manifestation of progressive pulmonary fibrosis. The distinction of PSE can be tricky, and they have actually been regarded as a variant of this type of emphysema [1]. However, the frequency of involvement of the lower lobes, the interrupted septae mentioned above, the frequent increase in size over time, and the association with other CT findings suggesting UIP are distinct from these lesions compared with PSE alone or AEF/SRIF cysts.

Pathologically, in the largest necropsy study about the topic [22], these cysts were described as destruction of the alveoli and dense fibrosis (Fig. 4c) of the walls that can be similar to that seen in SRIF/AEF, but the frequent presence of fibroblastic foci was a distinctive feature. Moreover, although these cysts do not contribute to the diagnosis of UIP, they were usually described as apposed to the honeycomb lesions of UIP. When in the lower lobes, they were adjacent to normal parenchyma and honeycombing. However, in the upper lobes, they were next to emphysematous parenchyma.

### Honeycombing

**Fig. 6:**
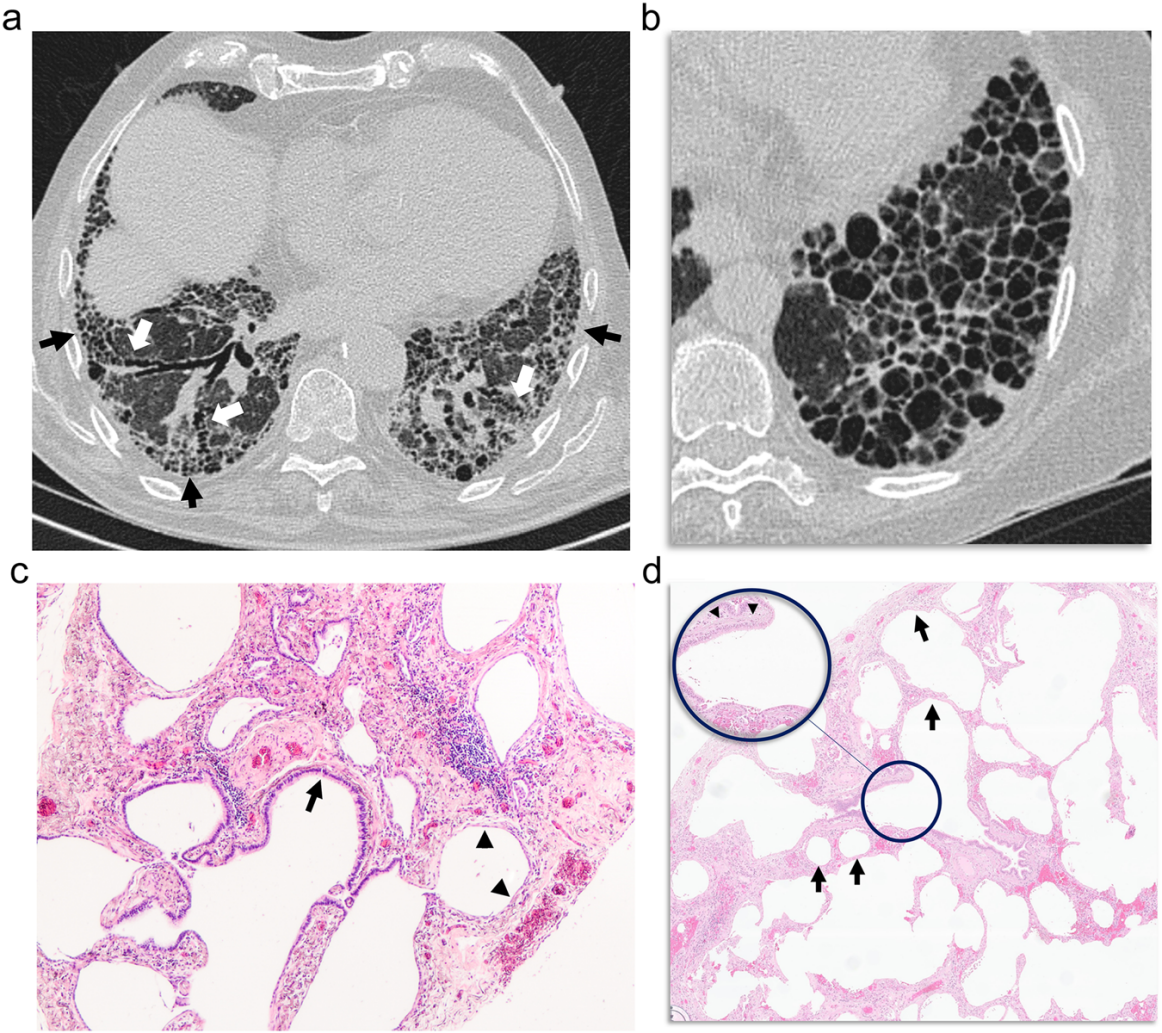
Honeycombing. **a** CT shows basal fibrosis with small honeycombing cysts (black arrows) consisting of rounded cystic lesions clustered in the subpleural region associated with traction bronchiectasis (white arrows). **b** Bigger, thick-walled honeycombing cysts sharing their walls are seen. **c** Pathologically, in samples from other patients, honeycombing is characterized by enlarged airspaces surrounded by fibrosis and lined by bronchiolar (arrow) or hyperplastic alveolar epithelium (arrowheads). **d** The transition from bronchiolectasis, with bronchial epithelium and a uniform muscular layer (arrowheads in the inset magnification), to respiratory-lined cysts (arrows) is shown

Honeycombing has been defined radiologically as well-defined, rounded cystic structures, typically clustered in the subpleural region (Fig. 6a, b) as a consequence of destruction of lung parenchyma with loss of architecture. Although typically defined as several layers of cysts, a single layer is used for diagnosis, provided other signs of fibrosis are present [26, 27]. Distinction of honeycombing from emphysema and other cystic lesions associated with smoking ILD is challenging [28, 29], with two large studies showing only a moderate interobserver correlation for the diagnosis of honeycombing, even among experienced radiologists [30, 31]. As a matter of fact, it has been described that honeycombing cysts are bigger in heavy smokers compared with non-smokers or patients with a lower smoking habit [16].

Pathologically, honeycombing is characterized by enlarged airspaces surrounded by fibrosis and lined by bronchiolar or hyperplastic alveolar epithelium (Fig. 6c, d) [26, 32]. In a study [33], honeycombing at CT has been correlated pathologically in explants mainly with respiratory-lined cysts and bronchiolectasis, the latter reflecting the continuum between bronchiolectasis and honeycombing as the hallmark findings of fibrosis at CT (Fig. 6c, d). The distinction of honeycombing cysts from coexisting cystic emphysematous lesions in fibrotic areas may be difficult for pathologists. In one study, honeycombing cysts were differentiated from emphysematous cysts by identifying columnar epithelial cells lining the inner surfaces of cysts [34].

### Smoking-related diffuse cystic lung disease

**Fig. 7:**
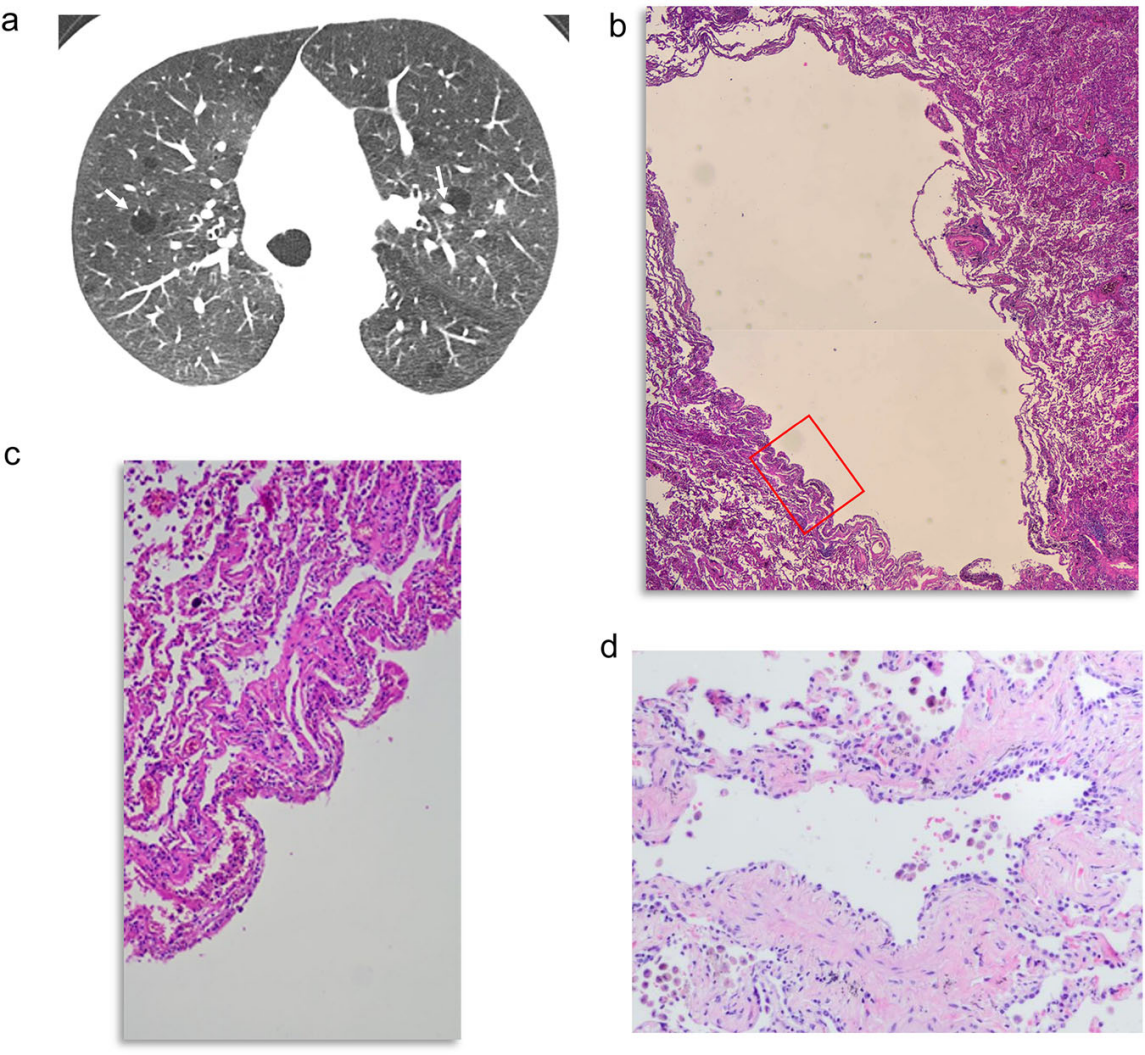
Smoking-related diffuse cystic lung disease in a 56-year-old smoker who underwent a left upper lobectomy for lung cancer. **a** At CT, multiple cysts, some of them perivascular (white arrows), with thin, regular walls, are seen. **b** Photomicrograph from the lobectomy specimen shows one of these cysts corresponding to airspaces surrounded by alveolar walls of normal thickness, as shown in the magnification view of this case in **c**. **d** Respiratory bronchiolitis with peribronchiolar fibrosis was also evident in other sites in this patient

Although we lack evidence to consider it as a distinctive pathological entity, smoking-related diffuse cystic lung disease was defined by Gupta et al [35] in a series of four female smokers presenting with multiple rounded pulmonary cysts with the suspicion of lymphangioleiomyomatosis. These cysts were frequently perivascular, showing eccentric vessels and septations (Fig. 7a).

Pathologically, the cysts corresponded to airspaces surrounded by alveolar walls of normal thickness (Fig. 7b, c). In all the cases, alveolar destruction consistent with emphysema and chronic bronchiolitis with features of respiratory bronchiolitis (RB) (Fig. 7d) were seen [35].

### Lung cysts in Langerhans cell histiocytosis (LCH)

**Fig. 8:**
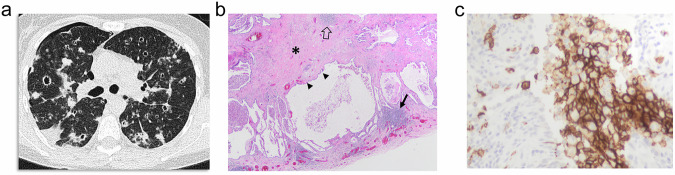
LCH in a 41-year-old female smoker. **a** CT shows multiple variable-shaped and sized cysts with thick walls. A right-sided pneumothorax is seen. **b** The photomicrograph corresponding to the right upper lobe depicts a cystic space next to a nodule formed by aggregates of Langerhans cells (arrow). The walls of the cyst in this case are partially formed by bronchial epithelium (arrowheads). Other nodules (open arrow) and dense fibrosis (asterisk) are also seen. **c** Langerhans cells in the nodules are confirmed by positivity to CD1a

Lung cysts and cavitated nodules are part of the evolution of LCH. This condition is characterized by proliferation of peribronchial infiltrates of Langerhans cells that progress to cavitation of the nodules and destruction of the lungs, with end-stage LCH sometimes mimicking an extensive CLE. This poses a diagnostic challenge for both radiologists and pathologists [36]. Nodules and thick-walled cysts (Fig. 8a) are manifestations of the disease at the earlier stages, followed by the development of thin-walled cysts that are variable in size and may coalesce into irregular cysts [37–39]. Final stage manifestation is that of emphysematous lesions, sometimes with irregular walls that are difficult to differentiate from emphysema with fibrous walls.

Histologically, the cystic spaces appear at the periphery of the nodules (Fig. 8b) secondary to traction on surrounding alveolar walls or airways. Nodules are formed by aggregates of Langerhans cells confirmed by positivity for CD1a (Fig. 8c), and they are accompanied by fibrosis (Fig. 8b) [36].

### Cysts in desquamative interstitial pneumonia (DIP)

**Fig. 9:**
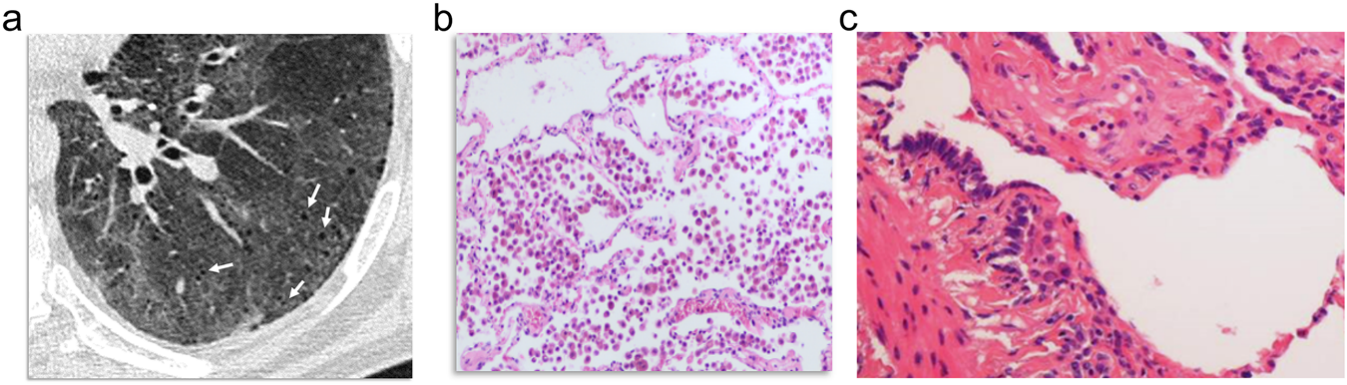
DIP in a 59-year-old male smoker. **a** CT shows basal diffuse ground-glass opacities and tiny rounded cysts (arrows). **b** The photomicrograph corresponding to the left lower lobe shows widespread pigmented macrophages occupying the distal airspaces. In this case **c**, the small cystic structures corresponded to alveolar ducts and were accompanied by fibrosis

DIP is characterized by bilateral ground-glass opacities mainly located in the basal parts of the lung, and it is often associated with fine reticulation and occurs most frequently in patients with exertional dyspnoea and cough [40]. Tiny 2–4 mm round cysts can be seen at CT (Fig. 9a) in a range from one third [40, 41] to 75% of the cases with DIP [42].

Recently, the update of the international multidisciplinary classification of the interstitial pneumonias proposed by the European Respiratory Society and the American Thoracic Society changed the term “DIP” to “alveolar macrophage pneumonia” [43].

Pathologically, DIP or alveolar macrophage pneumonia, represents a part of the spectrum of accumulation of pigmented macrophages in smokers that ranges from RB, in which macrophages are restricted to the lumens of respiratory bronchioles and alveolar ducts to more extensive diffuse accumulation of pigmented macrophages within most of the distal airspace of the lung (Fig. 9b) [3]. However, DIP has also been described in non-smokers [40]. The cysts have been regarded as fibrotic cysts that differed from honeycombing cysts [42, 44], but also as dilatation of alveolar ducts (Fig. 9c) and bronchiectasis [42].

## Fibrosing lung diseases and cystic lung lesions in smokers

From a pathologist’s perspective, fibrosis is not an infrequent finding in the lungs of individuals with a smoking history. A number of pathologic conditions can exhibit different grades and types of fibrosis. Pathology of smoking-related ILD is characterized by the combination and overlap of histological findings, occasionally giving rise to confusing terminology, with different names for similar findings that make boundaries between some entities markedly difficult to define and somewhat subjective, so that the final diagnosis can be influenced by the observer and conditioned by the sample available [2, 3]. For example, in the case of the UIP pattern, regardless of the existence of well-defined criteria [25], a high level of interobserver variability in the diagnosis is observed between pathologists [45, 46]. Moreover, pathologic and radiologic evolution occurs over time, thus making the issue much more complex. This is the case in the development of signs of fibrosis with honeycombing in a proportion of previously non-fibrotic DIP [47].

Among the terms used in the literature to refer to different types of fibrosis in smokers, we can find RB with fibrosis [48], RB-associated interstitial lung disease (RBILD) with fibrosis [49], AEF [18], emphysematous fibrosis [21], SRIF [50], fibrotic DIP [3], non-specific interstitial pneumonia (NSIP) [51], fibrosis associated to LCH, UIP, and even just “interstitial fibrosis” not otherwise specified [21]. Given this variety of terms, an analysis of the literature for a radiologic–pathological correlation is complex. Perhaps radiologist should have in mind the statement by some pathologists in the case of DIP and NSIP, that affirmed that “in the end, the distinction between DIP and fibrotic NSIP is often arbitrary” and the most important role of pathologists should be to underscore that the patient has a smoking-related fibrotic lung disease that is not UIP [3]. The description of these types of fibrosis, together with other radiologic and pathologic terms used in this manuscript, are in Table 3.

**Table 3:** Glossary of radiologic and pathologic terms in ILD in smokers

Term	Description
AEF	Pathologic term described by Kawabata et al [18] characterized by multiple thin-walled cystic lesions, histologically intensive hyalinized fibrosis, and a bronchiolocentric location.
Alveolar macrophage pneumonia	It is a pathologic term suggested in the 2025 update of the ERS/ATS classification of interstitial pneumonias as a replacement for the term “DIP”.
CPFE	This term was initially used by Cottin et al [14] who described a clinical-radiological syndrome based on the chest computed tomography findings of emphysema of the upper zones and diffuse parenchymal lung disease with fibrosis of the lower zones of the lungs in patients with abnormal spirometry, severe impairment of gas exchange, high prevalence of pulmonary hypertension, and poor survival. In a recent research statement by the ATS/ERS/JRS/ALAT [1], the heterogeneity of definitions and diagnostic criteria used for this term in the literature is noted, and they propose a clinical definition of “CPFE clinical syndrome” and a research definition of CPFE characterized by the coexistence of fibrosis and emphysema.
CLE	Pathologic term described as permanent enlargement of airspaces distal to the terminal bronchiole, accompanied by the destruction of their walls, without obvious fibrosis [10]. This term has been adopted by radiologists to designate its radiologic counterpart, consisting of small, well-defined or poorly defined areas of low attenuation surrounded by normal lung.
DIP	This is a pathological term described as diffuse accumulation of numerous pigmented macrophages in alveolar spaces [40], usually accompanied by some degree of fibrosis or inflammation. It conforms to a pathologic spectrum that includes a variety of appearances in both the number and distribution of macrophages, and it may be considered a “pathologic finding” that overlaps with and takes part in other forms of smoking-related ILD.
Emphysematous fibrosis	Pathologic term used by Miller et al [21] defined as hyalinizing interstitial fibrosis present predominantly in association with airspace destruction and enlargement.
Honeycombing	Pathologic term defined as enlarged airspaces lined by bronchiolar or hyperplastic alveolar epithelium and often containing mucin and inflammatory cells due to fibrosis in the parenchyma [26, 27]. Radiologically typical honeycombing is defined as clustered cystic airspaces with well-defined walls, usually presenting as multiple subpleural layers and associated with other signs of fibrosis [26, 27].
LCH	Pulmonary LCH is a rare histiocytic disorder that almost exclusively affects the lungs of smokers, characterized by nodules composed of a heterogeneous cell population including cells that exhibit the phenotype of Langerhans cells and mixed acute and chronic inflammatory cells [36].
NSIP	This is a type of ILD characterized pathologically by the presence of diffuse alveolar wall thickening by uniform fibrosis, with preserved alveolar architecture, mild interstitial inflammation, and no honeycombing or fibroblastic foci. The fibrotic form may resemble other fibrotic conditions associated with smoking, such as SRIF or fibrotic DIP; however, the inflammatory component and the lack of peribronchiolar or subpleural predominance favor the diagnosis of NSIP [3].
PSE	Pathologic term that refers to focal emphysematous destruction of the distal acinus next to the pleural surfaces. As for CLE, this term has been adopted by radiologists to designate its radiologic counterpart, characterized by foci of low attenuation in a subpleural distribution separated by interlobular septa [8, 13].
RB	This is a pathologic term defined by the presence of pigmented alveolar macrophages clustered within the lumens of respiratory bronchioles and peribronchiolar air spaces without significant inflammation or fibrosis [1]. It is an extremely common finding in the lungs of smokers, therefore, it can be present together with many other pathologic conditions in smokers.
RB-associated ILD with fibrosis	This is a pathologic condition described by Yousem [49] as RB having extensive paucicellular lamellar eosinophilic collagenous thickening of alveolar septa in a patchy, particularly subpleural distribution, and occurring in the presence of emphysematous change.
RB with fibrosis	This is a pathologic term suggested by Reddy et al [48], defined as localized patches of interstitial fibrosis mixed with emphysema that often radiate from a respiratory bronchiole to the pleura and are admixed with smoker’s macrophages.
Thick-walled cystic lesions	Thick-walled cystic lesions were defined radiologically by Inomata et al [22] as cysts measuring at least 1 cm in diameter and delineated by a 1-mm-thick wall in an area of the lung where reticulation and/or honeycombing was evident on CT images. In this autopsy study [22], this type of cyst was defined pathologically as cysts with dense wall fibrosis and occasional fibroblastic foci surrounded by honeycombing and normal alveoli. These cysts appeared only in patients with combined pulmonary fibrosis with the UIP pattern and emphysema.
Thin-walled cysts	These cysts were defined by Kawabata et al [18] as a macroscopic feature of AEF consisting of multiple cysts slightly apart from the pleura and occurring in AEF. Watanabe et al [16] described the radiologic appearance of these cysts as subpleural (but not abutting the pleura) thin-walled cystic lesions that did not affect the lung base. They were associated with the diagnosis of AEF.
Traction emphysema	This is a radiologic term coined in the research statement by the ATS/ERS/JRS/ALAT [1] to designate the thick-walled cystic lesions described above. This name was proposed because of its resemblance to the mechanism of formation of traction bronchiectasis.

*CPFE* combined pulmonary fibrosis and emphysema, *DIP* sesquamative interstitial pneumonia, *NSIP* non-specific interstitial pneumonia, *RB* respiratory bronchiolitis, *UIP* usual interstitial pneumonia

Although all these forms of fibrosis may eventually appear in some patients fulfilling the criteria to be considered to belong to the “syndrome of combined pulmonary fibrosis and emphysema” [1], their clinical behavior is highly variable. Clinically, when dealing with ILD in smokers, what is relevant is whether it is progressive or, on the contrary, it is expected to exhibit a stable course.

Considering this scenario, the role of the radiologist should be to try to characterize the underlying ILD present and specifically to correctly identify signs associated with progression of pulmonary fibrosis, mainly traction bronchiectasis and honeycombing, that greatly determine both the risk of progression and the prognosis [25, 52–54]. At this point, distinction of honeycombing cysts from emphysema and other types of cysts described above is essential to avoid the wrong radiologic diagnosis that may lead to unnecessary therapy with antifibrotic drugs. Discussing the pathological and radiologic characteristics of all the smoking-related ILD is beyond the scope of this article, but we should pay attention to two forms of fibrosis that frequently appear in smokers and exhibit cystic lung lesions: UIP and SRIF, together with AEF and RB-ILD, which have important pathologic similarities with SRIF. Although UIP and these three types of fibrosis may coexist in the same patient [18], their distinction is of major importance due to their different prognosis and management.

**Fig. 10:**
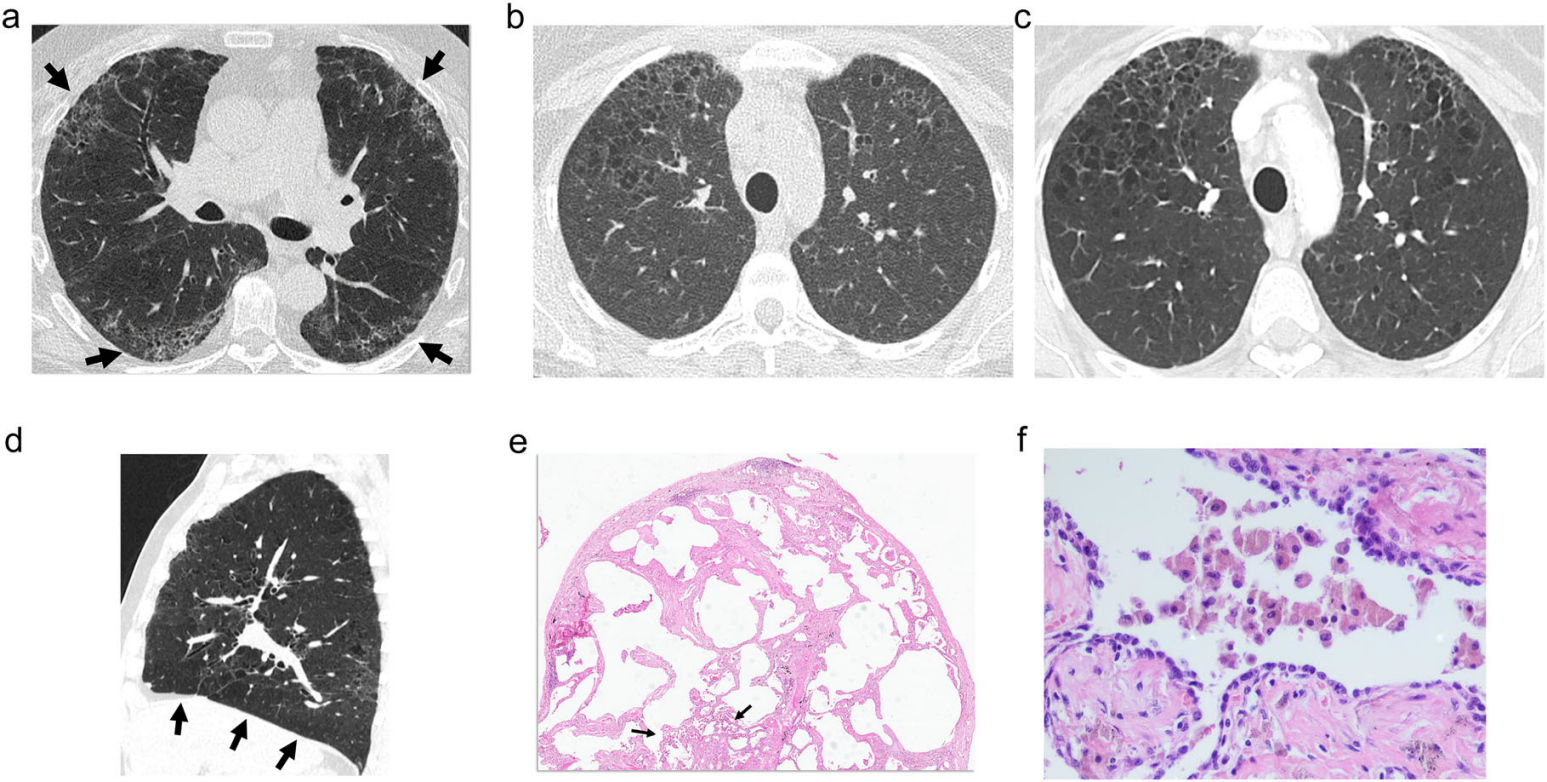
SRIF. Distribution of abnormalities in CT are shown in three different cases of pathologically confirmed SRIF: in **a** CT shows ground-glass, reticulation and some small cystic lesions in the periphery of the anterior aspects of the upper lobes and the posterior region of the upper segments of the lower lobes (arrows) mimicking “the four corners sign”; in **b** reticulation, emphysema and thin-walled cysts in the anterior upper lobes, that in a follow-up CT scan 15 years later (**c**) showed stability of findings without significant progression; and in **d**, sagittal reconstruction of the left lung showing how the findings are limited to the middle zones of the lung with sparing of the more basal diaphragmatic surface of the lungs (arrows). **e** At pathological examination corresponding to the upper segment of the left lower lobe of the patient in **a**, dense eosinophilic collagen thickening involves the alveolar septa and the subpleural area, together with some pigmented macrophages in the alveolar airspaces (arrows). **f** RB is shown with an accumulation of macrophages within the lumens of the respiratory bronchioles

SRIF (Fig. 10) is characterized by varying degrees of alveolar septal widening by hyalinized collagen deposition along with emphysema and RB (Fig. 10d, e). Fibrosis occurs both in subpleural and centrilobular parenchyma and surrounds enlarged airspaces of emphysema, but it also involves non-emphysematous parenchyma [50]. In 2006, Yousem et al [49] described “RB-associated ILD with fibrosis” as RB having extensive paucicellular lamellar eosinophilic collagenous thickening of alveolar septa in a patchy, particularly subpleural distribution. Finally, under the term of “airspace enlargement with fibrosis”, Kawabata et al [18] also described pathologic changes consisting of fibrous (frequently hyalinized) interstitium with structural remodeling, emphysematous change, and frequent bronchiolocentric location. All these three pathologic conditions were frequent in the lungs of smoker patients, appearing in 14 to 40% of subjects, and showed a good prognosis compared to patients with UIP, with little or no progression [18, 49, 50, 55] and a longer survival [28].

Regarding their radiologic manifestations, in a review of lung resections for nodular lesions [56], in two-thirds of the patients showing SRIF, there was no interstitial lung abnormality at CT. When CT showed any abnormality, patchy circumscribed areas of reticulation mixed with emphysema in a peripheral distribution were seen, as well as circumscribed ground-glass attenuation (Fig. 10a–d). The thin-walled cysts described above are the radiologic hallmark of AEF [16] and SRIF [20]. In a recent series [20], when SRIF was compared with UIP and emphysema, these cysts were the most useful distinguishing feature, together with the presence of subpleural ground-glass opacities associated with reticulation and smoking-related disease in the form of centrilobular ground-glass opacities in the upper lobes. Pathologically, these cysts represent emphysematous areas surrounded by fibrosis. The distribution of these findings characteristically involves the upper lobes and the superior segments of the lower lobes with sparing of the more basal zone (Fig. 10d) [16, 18, 48, 57]. In our experience, some cases show a distinctive involvement of the anterior upper lobes and superior segments of the lower lobes closely resembling the “four corners sign” distribution described in systemic sclerosis (Fig. 10a) [58]. A striking characteristic is the stability of the radiological findings over time [55], as shown in the case in Fig. 10b, c.

**Fig. 11:**
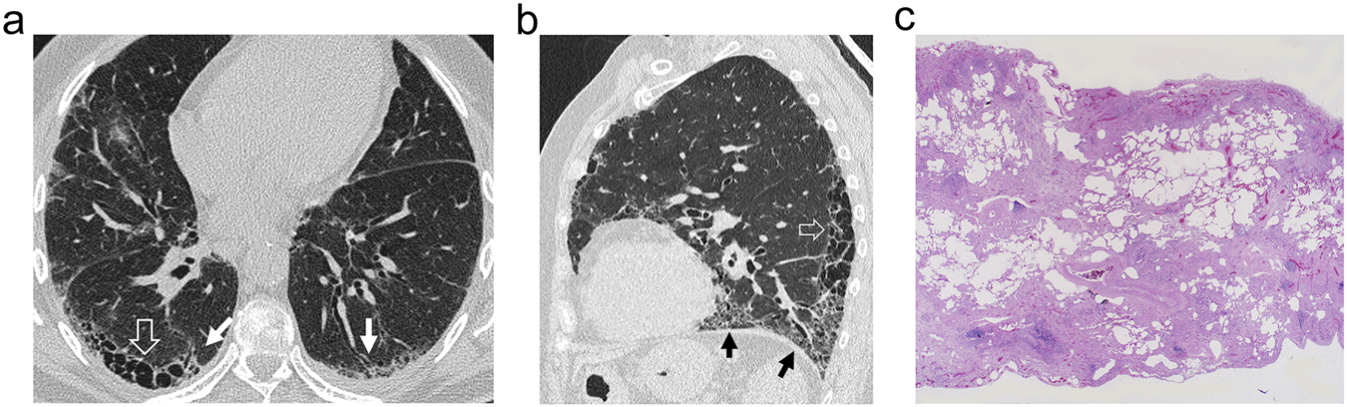
UIP. **a** CT shows subpleural cystic lesions in the right upper lobe (open arrow) and bilateral traction bronchiectasis (white arrows). **b** Distribution of abnormalities are shown in the sagittal section of the left lung, with involvement of the most basal lung next to the diaphragm (arrow), with cysts in the middle posterior zone. **c** Surgical lung biopsy of the left lower lobe demonstrated a UIP pattern with patchy, heterogeneous fibrosis

Fibrosis with UIP pattern (Fig. 11) carries a dismal prognosis in smokers. Its manifestation can be similar to that in non-smoker patients, but the combination of emphysema and fibrosis makes the differentiation of a UIP pattern more difficult to detect [29]. UIP is characterized by a patchy distribution of fibrosis with distortion of normal lung architecture, fibroblast foci, and honeycombing cysts. Radiologically, honeycombing can be particularly difficult to define and to differentiate from other cystic lesions when combined with emphysema. In the series mentioned above, comparing radiologic findings of SRIF, emphysema, and UIP [20], honeycombing was partly limited as a differentiating feature since it was present in 42% of cases with UIP, but also in 8.7% of SRIF. Traction bronchiectasis was even less useful due to its presence in all the cases of both UIP and SRIF. In contrast with SRIF and AEF, the UIP pattern most commonly affects the lower zones and the diaphragmatic surface of the lungs [18]. However, this distribution is not always evident, and in some cases, the boundary between emphysema and fibrosis is obscure at pathology, and both emphysema and fibrosis coexist in the upper and lower lobes [34]. Regarding the cysts known as traction emphysema, they can be associated with other findings of UIP (Fig. 5) [22], although several publications refer to them as characteristic of SRIF/AEF [1, 4, 24, 25, 59]. In our experience, although the fibrotic walls of these cysts may show dense eosinophilic collagen fibrosis like SRIF/AEF (Fig. 4c), they are frequently associated with other findings suggesting UIP elsewhere and with fibroblastic foci at pathological examination. This could explain the existence of cases with these cysts manifesting as acute exacerbation of ILD [59]. Given these discrepancies, radiologists should be reluctant to make a diagnosis of SRIF based solely on the presence of this type of cyst, and must consider other findings, mainly the distribution of the disease and the presence of honeycombing, subpleural ground-glass opacities, and reticulation or centrilobular ground-glass opacities [20]. When in doubt, radiologists should refrain from making a diagnosis of either SRIF or UIP, and histological confirmation or clinical, radiological, and functional follow-up should be recommended.

**Fig. 12:**
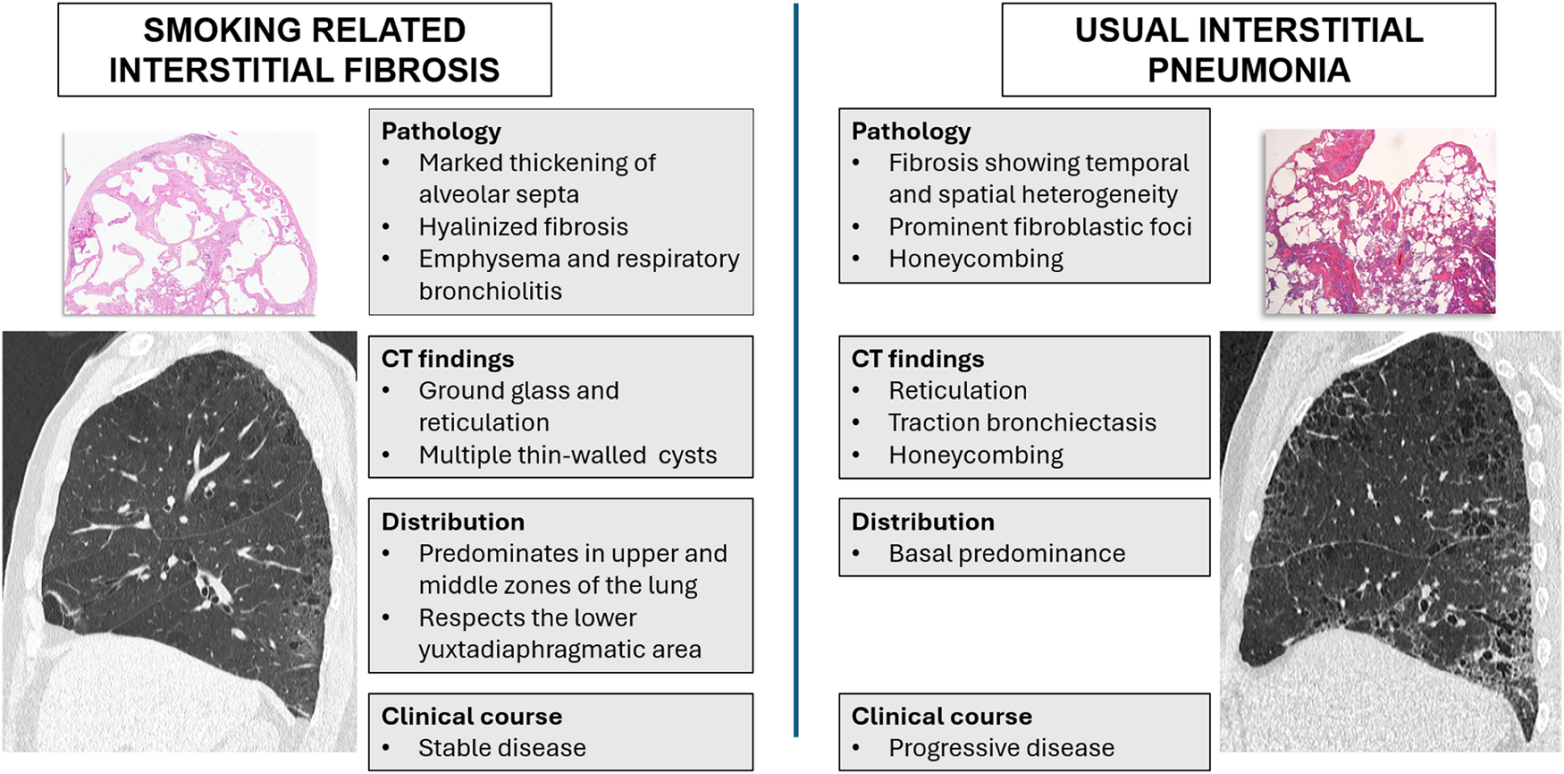
Summary of differences between SRIF and UIP

Differentiation of SRIF and UIP is critical to avoid inadequate management. Figure 12 shows a summary of the main differences between the conditions.

## Other considerations regarding cystic lung lesions in smokers

The evaluation of cystic lung lesions in smokers, like that of ILD, relies on an adequate technical examination. Either examinations with thick sections or an inadequate degree of inspiration could affect both the detection and characterization of these lesions.

Finally, the differential diagnosis of a cystic lung lesion in a smoker should take into consideration lung cancer associated with cystic airspaces [60]. The mechanism of formation of these lesions is varied and includes tumor growth in the wall of a preexisting cystic lesion, lepidic growth of adenocarcinoma in a background emphysematous lung parenchyma, and a check-valve mechanism obstructing a small airway leading to air trapping. In contrast with the rest of cystic lesions, lung cancer associated with cystic airspaces are usually a unique lesion with varied appearance, but most frequently presents as a cystic lung lesion with nodular wall thickening [60, 61].

In conclusion, radiologic diagnosis of cystic lung lesions in smokers is challenging. By discussing their radiologic–pathological correlation, we try to point to clues for a better understanding of these lesions. In Fig. 1 of the supplementary material, we provide readers with a flowchart proposing a radiologic diagnostic approach to these lesions.

## Supplementary information

ELECTRONIC SUPPLEMENTARY MATERIAL

## Data Availability

All data and materials presented were from the authors‘ hospital and daily practice.

## Abbreviations

AEF: Airspace enlargement with fibrosis
CLE: Centrilobular emphysema
CPFE: Combined pulmonary fibrosis and emphysema
DIP: Desquamative interstitial pneumonia
ILD: Interstitial lung disease
LCH: Langerhans cell histiocytosis
NSIP: Non-specific interstitial pneumonia
PSE: Paraseptal emphysema
RB: Respiratory bronchiolitis
SRIF: Smoking-related interstitial fibrosis
UIP: Usual interstitial pneumonia
