# Supplementary material for: Understanding cystic lung lesions in smokers with interstitial lung disease: radiologic–pathological correlation
Source: Insights Imaging. 2025 Sep 17;16:196. doi: 10.1186/s13244-025-02074-7 (PMC12443667; doi:10.1186/s13244-025-02074-7)
Supplement: Supplementary file 1 — ELECTRONIC SUPPLEMENTARY MATERIAL [file 13244_2025_2074_MOESM1_ESM.pdf]

# Understanding cystic lung lesions in smokers with interstitial lung disease: radiologic-pathological correlation

## ELECTRONIC SUPPLEMENTARY MATERIAL

Insights Imaging (2025) Arenas-Jiménez JJ, Aranda I, Shalygina S, Alenda C, Ferrandez-Ferrandez D, García-Garrigós E.

Figure S1. Flowchart of the differential diagnosis of aerated and cystic lung lesions in smokers (DIP: desquamative interstitial pneumonia).

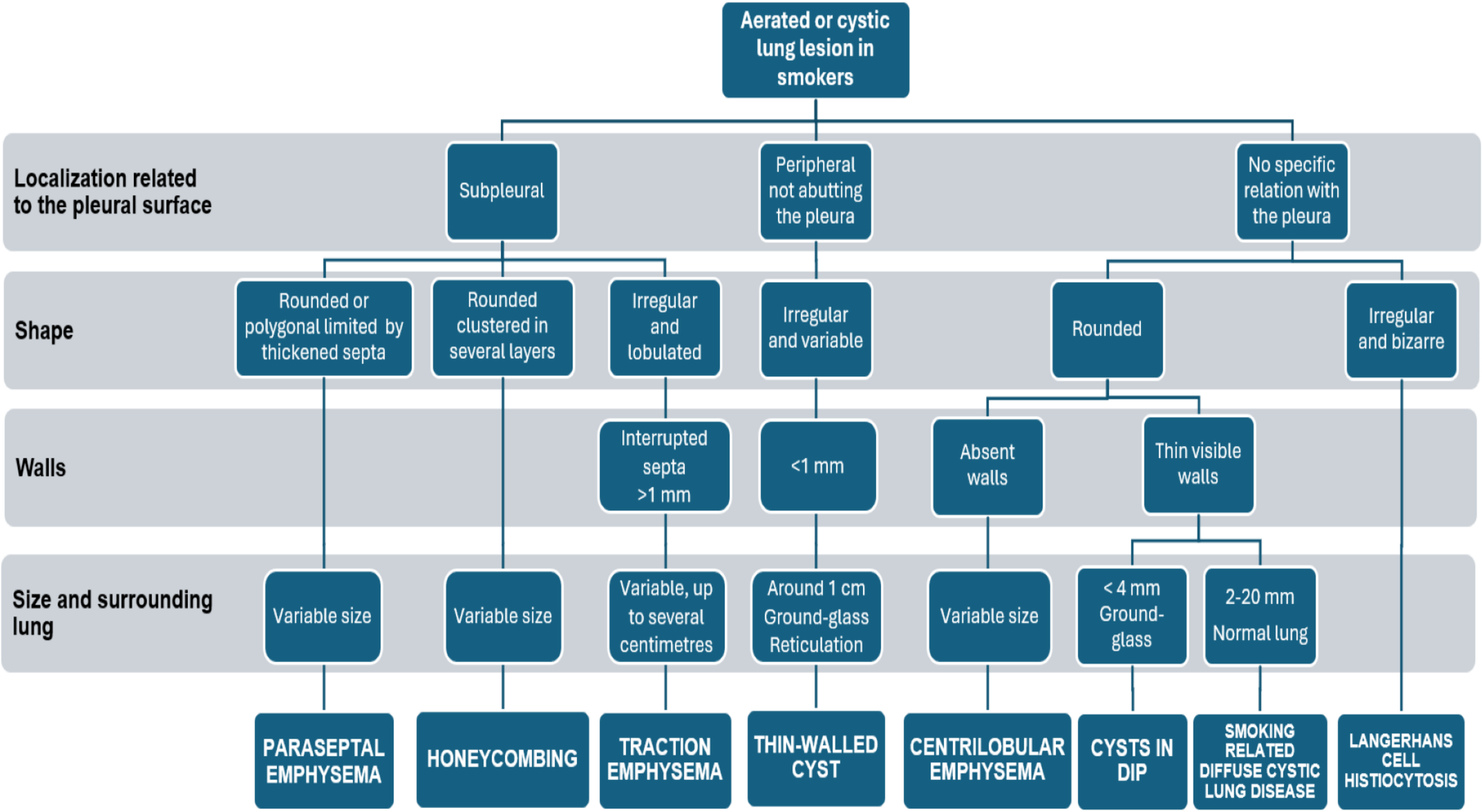


Insights Imaging (2025) Arenas-Jiménez JJ, Aranda I, Shalygina S, Alenda C, Ferrandez-Ferrandez D, García-Garrigós E.
